# Factors associated with occupancy of pharmacist positions in public sector hospitals in Uganda: a cross-sectional study

**DOI:** 10.1186/s12960-016-0176-x

**Published:** 2017-01-05

**Authors:** Thomas Ocwa Obua, Richard Odoi Adome, Paul Kutyabami, Freddy Eric Kitutu, Pakoyo Fadhiru Kamba

**Affiliations:** 1 Department of Pharmacy, School of Health Sciences, Makerere University, P.O. Box 7072, Kampala, Uganda; 2 Division of Pharmaceutical Services, Ministry of Health, Plot 6, Lourdel Road, P.O. Box 7272, Kampala, Uganda

**Keywords:** Uganda, Public hospital pharmacist, Attraction, Retention, Human resources for health

## Abstract

**Background:**

Pharmacists are invaluable resources in health care. Their expertise in pharmacotherapy and medicine management both ensures that medicines of appropriate quality are available in health facilities at the right cost and are used appropriately. Unfortunately, some countries like Uganda have shortage of pharmacists in public health facilities, the dominant providers of care. This study investigated the factors that affect the occupancy of pharmacist positions in Uganda’s public hospitals, including hiring patterns and job attraction and retention.

**Methods:**

A cross-sectional survey of 91 registered pharmacists practicing in Uganda and desk review of records from the country’s health care worker (HCW) recruiting agency was done in the months of May, June, and July, 2016. Pharmacist interviews were done using self-administered structured questionnaire and analyzed by descriptive statistics and chi-square test.

**Results:**

Slight majority (53%) of the interviewed pharmacists work in two sectors. About 60% of the pharmacists had ever applied for public hospital jobs. Of those who received offers (*N* = 46), 30% had declined them. Among those who accepted the offers (*N* = 41), 41% had already quit. Meanwhile, the pace of hiring pharmacists into Uganda’s public sector is too slow. Low socio-economic status of family in childhood (*χ* ^2^ = 2.77, *p* = 0.10), admission through matriculation and diploma scheme (*χ* ^2^ = 2.37, *p* = 0.12), internship in countryside hospitals (*χ* ^2^ = 2.24, *p* = 0.13), working experience before pharmacy school (*χ* ^2^ = 2.21, *p* = 0.14), salary expectation (*χ* ^2^ = 1.76, *p* = 0.18), and rural secondary education (*χ* ^2^ = 1.75, *p* = 0.19) favored attraction but in a statistically insignificant manner. Retention was most favored by zero postgraduate qualification (*χ* ^2^ = 4.39, *p* = 0.04), matriculation and diploma admission scheme (*χ* ^2^ = 2.57, *p* = 0.11), and working experience in private sector (*χ* ^2^ = 2.21, *p* = 0.14).

**Conclusions:**

The pace of hiring of pharmacists into Uganda’s public health sector is too slow and should be stepped up. Besides work incentives, affirmative action to increase admissions into pharmacy degree training programs through matriculation and diploma schemes and for children with rural childhoods should be considered.

**Electronic supplementary material:**

The online version of this article (doi:10.1186/s12960-016-0176-x) contains supplementary material, which is available to authorized users.

## Background

Effective health care delivery necessitates that medicines of the right quality and price are available to patients at the right time, the right medicines in the right doses are prescribed by clinicians, and the medicines are used appropriately. In reality, over 50% of prescriptions are inappropriate, over 50% of prescribed medications are administered incorrectly, and 20% of medicines in developing countries are of poor quality [1]. Mitigating this requires adherence to standard treatment guidelines by prescribers; personnel with expertise in medicine quality assurance, supply chain management, prescription analysis, professional dispensing, and enforcement of appropriate medicines use; and collaboration among different professional cadres in health care teams [2]. In health care teams, pharmacists are the professionals equipped with these competences. Pharmacists’ curricula extensively cover the physicochemical and pharmaceutical properties of medicines, pharmaceutical analysis, medicine supply management, dispensing, pharmacotherapeutics, pharmacoeconomics, and pharmaceutical care [1, 3, 4].

When utilized adequately in health care teams, pharmacists have proven beneficial in improving patients’ health outcomes. For instance, the involvement of pharmacists in clinical decisions has been shown to mitigate clinicians’ prescription errors [5]. In the United States of America, pharmacist interventions at a hospital not only reduced medication costs by 41% but also reduced medication errors by 66% [6]. A high rate of medication errors predisposes patients to therapeutic failure, adverse drug effects (ADEs), antimicrobial resistance, high treatment costs, poverty, and death. Inappropriate medicine use is costly to public health. The annual cost of inappropriate medicine use to the United Kingdom of Great Britain and Northern Ireland is 466 million pounds while in the USA, the annual cost per hospital is USD 5.6 million [7].

Pharmacists are not adequately utilized in the health systems of some developing countries. For example, Uganda’s expansive public health system is severely underserved by pharmacists. Despite Ugandan universities graduating at least 75 pharmacists annually [8], only 31 pharmacists are currently employed in the public sector against a set establishment of 371 [8, 9]. Shortage of pharmacists in Uganda’s public health system is so pervasive that even services where medication counseling is invaluable such as antiretroviral therapy are undertaken by non-pharmacists in 99% of the facilities [10]. Unsurprisingly, dispensing practices in Uganda’s public health facilities are poor and only 24% of medicines dispensed to patients are adequately labeled [8].

In Uganda, 55% of health facilities are public [11] and provide free services. Given that the country’s population is predominantly poor [12] and unable to meet out-of-pocket health expenditures, deficiencies in the country’s public health sector deprives majority of quality health care. Thus, the occupancy of pharmacists’ positions available in Uganda’s public health system ought to be improved. Critically, the factors undermining the occupancy of public hospital pharmacist positions ought to be understood.

Three scenarios can exacerbate low occupancy of job positions in an organization, namely, inadequate hiring, low job attractiveness, and high staff attrition. In Uganda, all the three scenarios have been reported to be prevalent [13]. Understanding the contribution of each of these variables and associated factors to the low public hospital pharmacist job occupancy in Uganda is paramount.

People get attracted to and stay on or quit their jobs for various reasons, including financial rewards (pay), job security, status, further education and career advancement opportunities, recognition, responsibility, professional support, working conditions, supervision, relationship with management and peers, and desire for achievement [14]. Additionally, socio-demographics such as age, gender, marital status, geo-economic background of childhood, education, and internship, have been variously implicated in job decisions [15, 16]. These factors have been widely studied in lieu of health care worker (HCW) attraction, motivation, and retention, in both surveys and interventional studies. One survey of satisfaction with the profession among United Kingdom pharmacists found that poor remuneration is associated with dissatisfaction while the amount of responsibility and service to patients favor satisfaction [17]. Meanwhile, interventional studies indicate that financial incentives, opportunities for career development, education and training, and work environment predominantly result in positive outcomes [15, 18]. In Uganda, a survey of 641 HCWs covering the public and private not-for-profit sectors found salary to be a key determinant of job satisfaction, yet 89% of them were dissatisfied with their salary [19]. Finally, there are reports that the mode of instruction (problem-based learning versus lecture-based learning) during health professional training affects career choice and personnel competences [2, 20]. In problem-based learning (PBL), students lead their own learning guided by a well-defined problem and learning objectives, while in the lectures, teachers lead the learning [21]. Understanding these factors in the context of the Ugandan pharmacist is important in formulating appropriate mitigation.

This study examined the factors that affect the occupancy of pharmacist positions in public hospitals in Uganda. Specifically, the hiring patterns, attraction (expression of interest), retention, and the factors associated with attraction and retention of pharmacists in Uganda’s public hospitals were investigated.

## Methods

### Study design

A cross-sectional survey of registered pharmacists practicing in Uganda and desk review of records from the country’s HCW recruiting agency, the Health Service Commission (HSC), was conducted during the months of May, June, and July, 2016.

### Study setting

Uganda is a low-income country in East Africa [12] with a population of 37 million and per capita gross domestic product of USD 650 [22, 23]. Uganda’s per capita expenditure on health is USD 12, far below the World Health Organization (WHO) recommendation of USD 34 [9]. The country’s formal health system comprises 2911 public and 1567 private health facilities [24]. In 2015, there were 616 pharmacists practicing in Uganda, but only 31 were employed in the public sector compared to an approved establishment of 376 [9, 24]. Thus, most of Uganda’s pharmacists are in private practice, of which 70% are in the capital Kampala and adjacent towns [25].

### Sample size and sampling procedure

Study objectives required that the sample of pharmacists was drawn from both the public health sector and the non-public health sector (private practice, academia, manufacturing, and medicines regulation). Thus, stratified sampling was used. A sample size of 96 comprising all the 31 pharmacists employed in the public health sector and 65 non-public health sector pharmacists was targeted. The 31 public health sector pharmacists were a universal sample due to the small population and are largely employed at the Ministry of Health headquarters, the nation’s two national referral hospitals and its 14 regional referral hospitals [9]. The sample size of non-public health sector pharmacists was calculated from a population of 556 at a margin of error of 5 and 95% level of confidence using Cochran’s formulae [26]. Initially, random selection of non-public health sector pharmacists was attempted. However, due to very low response rate and rampant absenteeism of pharmacists in Uganda’s private pharmacies, sampling was switched to a convenience approach. In the convenience sampling, non-public health sector pharmacists were selected based on accessibility and offer/acceptance of appointment to fill the questionnaire on contact by the investigator.

### Data collection

A desk review guide (Additional file 1) and a structured self-administered questionnaire (Additional file 2) were used. The desk review guide was used to extract information on recruitment rates (vacancies advertised and filled) from HSC records. This guide covered the period 2004/2005 to 2013/2014. The questionnaire was used to obtain data on whether a pharmacist had ever applied for public hospital jobs, whether they had ever rejected such job offers, and whether those who had ever accepted these jobs were still in service, plus the factors that affect attraction and retention of pharmacists into these jobs. For educational/professional factors, the questionnaire asked about respondents’ education and professional preparation, including rural-urban orientation of education and internship institutions. For economic factors, the questionnaire asked respondents on the financial motivations of career choice. For reliability and validity of the responses, the questionnaire was reviewed by three experienced researchers from the Makerere University prior to data collection. Both e-mail and physical delivery of questionnaires by the researcher was used, generating 16 and 75 responses, respectively.

### Data analysis

Data was entered into CSPro version 6.1, cleaned and exported into STATA version 13 for analysis. Descriptive statistics, specifically percentages, were used to analyze the prevalence of attraction and retention, as well as the different factors that affect pharmacist attraction and retention. Chi square (*χ* ^2^) test, and for low frequencies, Fisher’s exact test, was used to assess the influence of these factors on attraction and retention of pharmacists into public hospitals.

## Results

Information on the attraction and retention of pharmacists in public sector hospitals and the factors that influence them were collected from 91 pharmacists out of a target sample size of 96, a response rate of 94.8%. Of the 91 respondents, only 19 (21%) were affiliated to the public health sector (public hospitals and Ministry of Health).

### Socio-demographic characteristics of respondents

Majority of the respondents were in the 26–35-year age bracket (51.7%), male (71.4%), married (71.9%), and working within 20 km of Kampala City (Kampala, Mukono, and Wakiso towns) (73.3%) (Table 1). About 53% of them had bachelor’s degrees and 37% had master’s degrees, and the rest had other postgraduate qualifications. About 60% of them had ever applied for a public hospital pharmacy vacancy.

**Table 1:** Socio-demographic characteristics of respondents

Characteristic	Respondent frequency *n* (%)
Age in years	
21–25	7 (8)
26–30	25 (28)
31–35	22 (24)
36–40	13 (14)
40+ years	24 (26)
Gender	
Male	65 (71)
Female	26 (29)
Marital status^a^	
Single	25 (28)
Married	64 (72)
Highest education level attained	
Bachelor’s degree	48 (53)
Postgraduate degree	43 (47)
Mode of learning used on pharmacy degree ^a^	
- Lectures (teaching)	42 (58)
- Problem-based learning	31 (42)
Geographical location of work station ^a^	
Kampala/Wakiso/Mukono	66 (73)
Rest of Uganda	24 (27)
Current geo-economic location of family ^a^	
Urban (capital city, other urban council)	41 (47)
Rural	46 (53)
Geo-economic setting of respondent’s primary school education ^a^	
Urban	20 (23)
Rural	67 (77)
Geo-economic setting of respondent’s secondary school education ^a^	24 (28)
Urban	63 (72)
Rural	

^a^The sample size (*N*) for some responses is less than 91 due to missed responses, which are inevitable in self-administered questionnaires. Any variation in *N* in subsequent tables is also due to missed responses

**Fig. 1:**
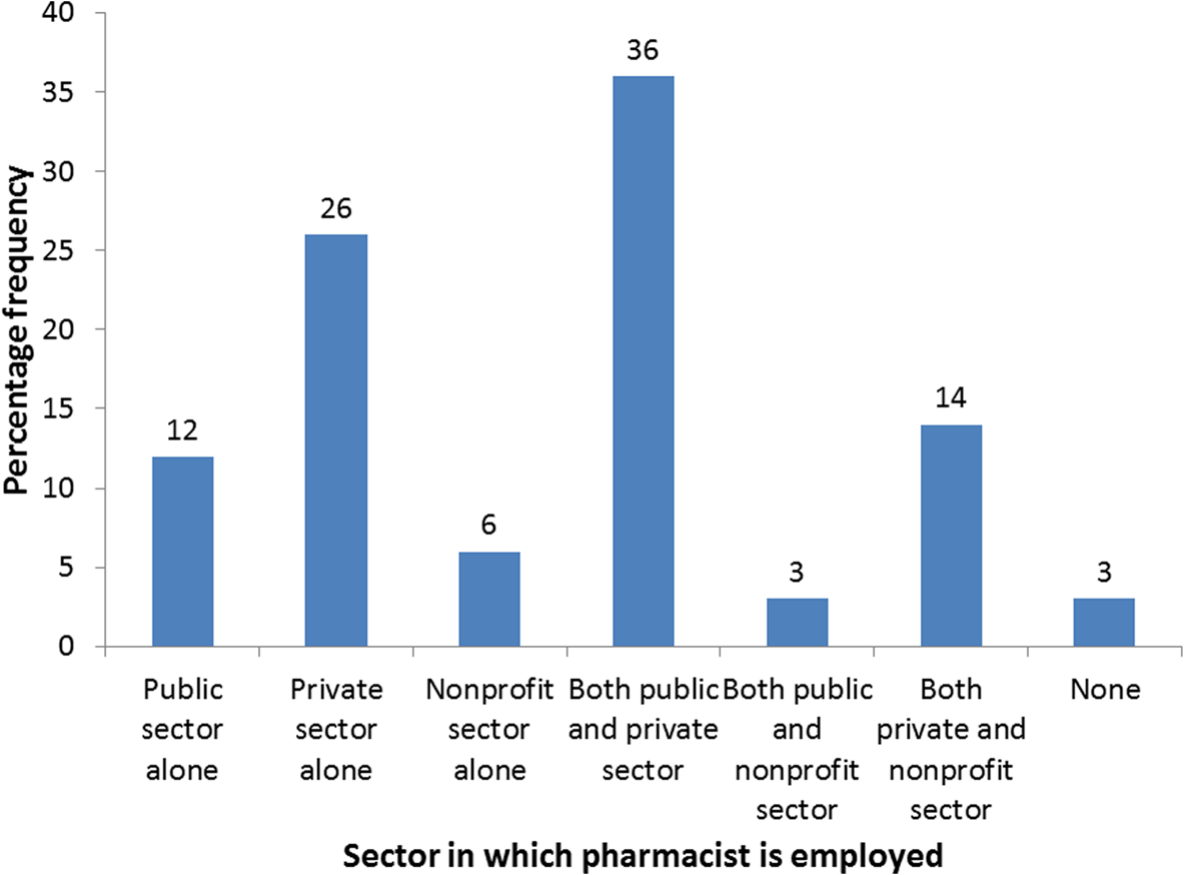
Distribution of respondents by sector of employment

Furthermore, a slight majority (53%) of the respondents are in dual practice, and for those in single practice, twice as many work in private practice compared to public sector (Fig. 1).

### Rates of attraction and retention of pharmacists in public hospitals

Attraction was measured as expression of interest (application for a job) and acceptance of offer of appointment while retention was measured by the rate of attrition (history of quitting) from public hospital job. Table 2 shows that only 60% of respondents had ever applied for a public hospital pharmacist job. Of those who had ever been offered appointment to public hospital jobs, 30% indicated that they declined the offer.

**Table 2:** Attraction and retention of pharmacists into public sector hospitals

Variable	Respondent frequency *n* (%)
Attraction	
Had ever applied for a public hospital pharmacist vacancy (*N* = 72)	43 (60)
Had ever accepted appointment as public hospital pharmacist (*N* = 41)	32 (78)
Had ever declined offer of appointment as public hospital pharmacist (*N* = 46)	14 (30)
Retention	
Had ever accepted offer of appointment as public hospital pharmacist but has since quit the job (*N* = 32)	13 (41)

Efforts were made to triangulate data on attrition and retention of pharmacists through desk review of records from the Human Resource Department (HRD) of Uganda’s Ministry of Health (MOH) and the HSC for the period 2004/2005 to 2013/2014. Unfortunately, no records were availed by the HRD. From the HSC, only records for 2010/2011 to 2013/2014 were accessed. Furthermore, the HSC records only showed the number of vacancies advertised per year and those filled (Additional file 1); it was not clear whether the vacancies were for previously unoccupied positions or for replacement of departed staff. These vacancies were also few compared to the human resource needs of the public health sector. In the 2013/2014 fiscal year, despite the availability of 345 pharmacist vacancies [9], only nine positions were advertised and filled by the HSC (Additional file 3).

### Factors associated with interest in public hospital pharmacist jobs

The association between interest in public hospital pharmacist jobs and the common determinants of career choice and job motivation (socio-demographic attributes, educational/professional factors, and economic factors) was determined. Among socio-demographic factors, age had the strongest association with interest in public hospital pharmacy jobs. Pharmacists 35 years and below were more likely to apply for public hospital jobs compared to those above 35. The association was however statistically insignificant (Table 3). Among economic factors, strong but statistically insignificant associations were recorded with the socio-economic status of the family during advanced level (A’Level) secondary education and salary expectations (Table 3). Pharmacists from families of low socio-economic status and those comfortable with salaries of less or equal to UGX 2 million were more likely to apply for public hospital jobs. Among educational/professional factors, strong but statistically insignificant associations were found with route of admission to pharmacy degree, geo-economic region in which respondent did pharmacist internship, prior working experience before starting pharmacy degree, and geo-economic location of secondary education, in decreasing order of significance (Table 4).

**Table 3:** Association of socio-demographic and economic factors with interest in public hospital pharmacist jobs

Variable	Ever applied for public hospital job	Never applied for public hospital job	*χ* ^2^	*p* value
A. Socio-demographic factors				
Age				
≤35 years	27	14	1.49	0.22
>35 years	16	15		
Gender				
Male	32	21	0.04	0.85
Female	11	8		
Marital status				
Single	13	7	0.39	0.53
Married	29	22		
Geo-economic location of family				
Urban	35	22	0.25	0.62
Rural	7	6		
Geo-economic location of pharmacist’s childhood				
Urban	25	16	0.10	0.75
Rural	16	12		
B. Economic factors				
Socio-economic status of family during A’Level education				
Low	23	10	2.77	0.10
Middle or high	18	18		
Wages are important in choice between public and private sector careers				
Yes	37	25	0.00	0.98
No	6	4		
A minimum salary of UGX 2,000,000 is needed to accept full time pharmacist job				
Yes	23	19	1.76	0.18
No	5	1		
Pharmacist has additional economic activities besides employment				
Yes	25	19	0.15	0.70
No	16	10

**Table 4:** Association of educational/professional factors with interest in public hospital pharmacist jobs

Variable	Ever applied for public hospital job	Never applied for public hospital job	*χ* ^2^	*p* value
Highest education level attained				
Bachelor’s degree	26	14	1.04	0.31
Postgraduate qualification	17	15		
Geo-economic location of secondary education				
Urban	30	23	1.75	0.19
Rural	12	4		
Geo-economic location of primary education				
Urban	25	18	0.51	0.48
Rural	18	9		
Route of admission to pharmacy degree training				
Direct entry (A’Level certificate)	30	24	2.37	0.12
Diploma/mature age entry	13	4		
Prior working experience before starting pharmacy degree				
Yes	20	8	2.21	0.14
No	22	19		
Sector in which respondent worked before starting pharmacy degree				
Public	9	3	0.35	0.55
Private	13	7		
Mode of learning during pharmacy degree				
Lectures (teaching)	19	17	0.18	0.67
Problem-based learning	14	10		
Geo-economic region in which respondent did pharmacist internship				
Kampala City	8	9	2.24	0.13
Rest of the country	35	17		

### Factors associated with rejection of offer of appointment as public hospital pharmacist

The association between history of rejection of public hospital pharmacist job offer and the determinants of career choice and job motivation was determined. Among socio-demographic factors, there were reasonable but statistically insignificant associations between gender and rejection of job offer and between marital status and rejection of job offer (Table 5). Females and the married were less likely to reject public hospital pharmacist job offers compared to the contrary. Among economic factors, in decreasing order of importance, there were strong but statistically insignificant associations between the socio-economic status of the family during A’Level secondary education and rejection of public job offer and between low pay and rejection of job offer (Table 5). Pharmacists whose families were of low socio-economic status during A’Level were more likely to reject public hospital job offers than those from middle-high income families, in contrast to the effect of the same factor on interest in public hospital jobs (Table 3). Among educational/professional factors, only weak and statistically insignificant associations with rejection of public hospital job offer were found (Table 6).

**Table 5:** Association of socio-demographic and economic factors with rejection of offer of appointment as public hospital pharmacist

Variable	Ever rejected job offer	Never rejected job offer	*χ* ^2^	*p* value
A. Socio-demographic factors				
Age				
≤35 years	9	20	0.01	0.91
>35 years	5	12		
Gender				
Male	12	22	1.45	0.23
Female	2	10		
Marital status				
Single	5	7	1.16	0.28
Married	8	24		
Geo-economic location of family				
Urban	12	26	0.02	0.87
Rural	2	5		
Geo-economic location of pharmacist’s childhood				
Urban	8	18	0.03	0.86
Rural	6	12		
B. Economic factors				
Socio-economic status of family during A’Level education				
Low	10	15	3.04	0.08
Middle or high	3	16		
Wages are important in choice between public and private sector careers				
Yes	11	28	0.60	0.44
No	3	4		
A minimum salary of UGX 2,000,000 is needed to accept full time pharmacist job				
Yes	8	15	0.09	0.76
No	2	5		
Rejected a public hospital pharmacist job offer due to low pay				
Yes	1	0	1.94	0.16
No	4	9		
Pharmacist has additional economic activities besides employment				
Yes	7	21	0.76	0.38
No	6	10

**Table 6:** Association of educational/professional factors with rejection of offer of appointment as public hospital pharmacist

Variable	Ever rejected job offer	Never rejected job offer	*χ* ^2^	*p* value
Highest education level attained				
Bachelor’s degree	7	18	0.15	0.70
Postgraduate qualification	7	14		
Geo-economic location of secondary education				
Urban	10	23	0.04	0.85
Rural	4	8		
Geo-economic location of primary education				
Urban	8	18	0.00	0.96
Rural	6	14		
Route of admission to pharmacy degree training				
Direct entry (A’Level certificate)	8	23	0.96	0.33
Diploma/mature age entry	6	9		
Prior working experience before starting pharmacy degree				
Yes	9	15	0.98	0.32
No	5	16		
Sector in which respondent worked before starting pharmacy degree				
Public	2	7	0.93	0.33
Private	7	10		
Mode of learning during pharmacy degree				
Lectures (teaching)	7	16	0.00	0.96
Problem-based learning	5	11		
Geo-economic region in which respondent did pharmacist internship				
Kampala City	3	9	0.35	0.55
Rest of the country	12	23		

### Factors associated with retention of pharmacists in public hospital jobs

The association between retention in public hospital jobs and the common determinants of career choice and job motivation was examined. Among socio-demographic factors, reasonable but statistically insignificant association was found between geo-economic location of pharmacist’s childhood and retention in public hospital job (Table 7). Pharmacists who spent their childhood in rural settings were slightly more likely to stay in public hospital jobs compared to the contrary. Among economic factors, involvement in additional economic activities (business, agriculture, et cetera) favored retention in public hospital jobs, though in a statistically insignificant manner. Pharmacists with additional economic activities were slightly more likely to stay in public hospital jobs compared to the contrary (Table 7). Among educational/professional factors, a strong and statistically significant association was found between the highest education level of pharmacist and retention in public hospital job. Pharmacists with only a bachelor’s degree were more likely to stay in their public hospital jobs relative to those with postgraduate qualifications. Strong but statistically insignificant associations were also found with route of admission into pharmacy degree, sector in which respondent worked before pharmacy degree and mode of learning during pharmacy degree. Pharmacists who were admitted to the degree through diploma and matriculation schemes were more likely to stay in public hospital jobs compared to those admitted directly from secondary school. Furthermore, pharmacists who worked in the private sector before admission to pharmacy degree were more likely to stay in public hospital jobs compared to those with pre-degree stints in the public sector. Lastly, pharmacists who underwent lecture-based training at pharmacy degree were slightly more likely to stay in public hospital jobs than those who used problem-based learning (PBL). Details of associations of educational factors with retention in public hospital jobs are in Table 8.

**Table 7:** Association of socio-demographic and economic factors with retention in public hospital pharmacist jobs

Variable	Still in public hospital service	Quit public hospital service	*χ* ^2^	*p* value
A. Socio-demographic factors				
Age				
≤35 years	13	8	0.16	0.69
>35 years	6	5		
Gender				
Male	13	9	0.00	0.96
Female	6	4		
Marital status				
Single	4	4	0.20	0.66
Married	13	9		
Geo-economic location of family				
Urban	16	3	0.36	0.55
Rural	11	1		
Geo-economic location of pharmacist’s childhood				
Urban	12	3	1.37	1.24
Rural	5	1		
B. Economic factors				
Socio-economic status of family during A’Level education				
Low	8	10	0.01	0.92
Middle or high	6	7		
Wages are important in choice between public and private sector careers				
Yes	17	2	0.17	0.68
No	11	2		
A minimum salary of UGX 2,000,000 is needed to accept full time pharmacist job				
Yes	7	1	0.09	0.76
No	11	1		
Quit hospital pharmacist job due to low pay				
Yes	2	4	0.39	0.53
No	4	4		
Pharmacist has additional economic activities besides employment				
Yes	14	4	1.30	0.25
No	7	5

**Table 8:** Association of educational/professional factors with interest with retention in public hospital pharmacist jobs

Variable	Still in public hospital service	Quit public hospital service	*χ* ^2^	*p* value
Highest education level attained				
Bachelor’s degree	13	4	4.39	0.04
Postgraduate qualification	6	9		
Geo-economic location of secondary education				
Urban	14	5	0.06	0.78
Rural	9	4		
Geo-economic location of primary education				
Urban	9	9	0.04	0.83
Rural	6	7		
Route of admission to pharmacy degree training				
Direct entry (A’Level certificate)	11	8	2.57	0.11
Diploma/mature age entry	11	2		
Prior working experience before starting pharmacy degree				
Yes	11	7	0.68	0.41
No	6	7		
Sector in which respondent worked before starting pharmacy degree				
Public	2	10	2.21	0.14
Private	3	3		
Mode of learning during pharmacy degree				
Lectures (teaching)	9	8	1.10	0.30
Problem-based learning	8	3		
Geo-economic region in which respondent did pharmacist internship				
Kampala City	5	14	0.54	0.46
Rest of the country	2	11		

## Discussion

### Public sector versus private sector distribution of pharmacist employment

A slight majority of Uganda’s pharmacists are engaged in dual public and private practice. Except for pharmacists in manufacturing who are restricted to one job, Uganda’s pharmacist practice guidelines permit dual practice [27]. A previous study of health professional trainees showed that opportunities for dual practice are highly valued by pharmacy students in choosing work between rural and urban locations [28]. Hence, the prevalence of dual practice among Uganda’s pharmacists was not surprising. However, while dual practice helps maximize the use of scarce human resources, it can be detrimental to staff performance and is prohibited in some jurisdictions such as Canada [29]. Because of competition for time, dual practice can distract public servants from their primary responsibilities and exacerbate absenteeism. At worst, dual practice exacerbates pilferage of health supplies from public health facilities [29, 30]. Thus, health systems ought to monitor and regulate dual practice.

### Attraction and retention of Uganda’s public hospital pharmacists

There is significant baseline interest of Ugandan pharmacists in public hospital jobs. However, the high interest in the jobs is not translated into actual employment and retention in service as the proportion of pharmacists who have declined pubic hospital job offers and those who have quit their positions were significantly high, at 30 and 41%, respectively. Staff turnover is costly to organizations in terms of production, recruitment, training/mentoring, reputation, employee motivation, and customer satisfaction [31]. Therefore, conditions to maximize attraction and retention of pharmacists in Uganda’s public hospitals ought to be prioritized.

### Factors that attract pharmacists into public hospital jobs in Uganda

This study found that younger pharmacists were more likely to apply for public hospital jobs than older ones. A study of motivation of primary HCWs in Kenya found that majority were not older than 35 years [32]. Another study in Ghana found that majority of the country’s HCWs is under 40 years [33]. Thus, it appears that there is more interest in public sector jobs early in health professional careers than later.

A recent review indicates that the geo-economic setting of both a health professional’s early life and education impacts on interest to work in rural areas [15]. In fact, the WHO recommends that nations reserve slots for students from rural backgrounds in health training institutions if they are to boost HCW retention in deprived areas [20]. We found a sizable influence of geo-economic setting of secondary school education and pharmacist internship on application for public hospital jobs. Therefore, interventions that push more rural kids into pharmacist training have potential to improve the number of applications for public hospital jobs. Such interventions could include performance improvements in rural secondary schools and affirmative admissions into pharmacy programs at universities.

The favorable association between joining pharmacy degree as a mature/working class student and interest in public hospital jobs is worth considering because most HCWs in developing countries are of pre-degree education. In Kenya, a recent survey found that 80% of primary HCWs are of pre-degree education [32]. Therefore, building graduate HCWs through facilitation of existing staff to upgrade could be superior to recruitment from the general market.

### Factors that enhance retention of pharmacists in public hospital jobs in Uganda

We found that acquisition of postgraduate degree by pharmacists is counterproductive to retention in public hospital service. A recent study of medical doctors in a South African hospital found that prospects for career advancement, particularly opportunities for senior posts and the presence of an academically stimulating environment are fundamental desires [31]. Another study, in Kenya, also found career development as a key determinant of HCW motivation [33]. Plus, adequate pay is a key retention factor of HCWs in their jobs [15, 18]. Postgraduate training of staff impacts on all these factors. Not only does it empowers employees with new skills but it also raises their academic standing and life desires, including expectations for job responsibility, compensation, and societal status. It is therefore possible that the work conditions for public hospital pharmacists in Uganda fall below what comes with new academic status, leading to job dissatisfaction. We did not probe into the interventions in place to stem flight of serving staff following postgraduate training. However, tools such as bonding and stiff penalty for violation of the bond have been efficacious in some developing countries and could be explored by others, alongside motivational interventions. In Sri Lanka, which trains all its specialist doctors in developed countries, bonding and freedom to do private practice in off hours has helped in keeping emigration at 10% [34]. By and large, the specific reasons for flight of pharmacists with postgraduate degrees from Uganda’s public hospitals ought to be investigated to enable intelligent interventions.

Consideration should also be given to the other factors that were favorable to retention of pharmacist in public hospital jobs in Uganda, namely, admission into pharmacy training via diploma and matriculation, prior working experience in private sector, lecture rather than PBL instruction, rural childhood of pharmacist, and engagement in other economic activities. Similar to attraction, the Government of Uganda could uplift retention of hospital pharmacists by facilitating serving staff to obtain pharmacy degrees, a novel finding. The favorable associations between prior experience of pharmacists in the private sector and retention in public hospitals and between lecture-based education and retention were quite intriguing. In fact, it appears that joining public service after exploration of alternatives narrows the decision space and promotes career stability.

Besides impacting positively on learning and communication/interpersonal skills, no differences in the career patterns of graduates from the PBL and lecture-based systems are conceivable [21]. Hence, the favorable association between lectures and retention in public service was somewhat strange, though not surprising because previous studies have reported contrasting influences of PBL and lectures on rural medical practice, with lecture products posting higher odds of choosing rural-based careers [21].

The WHO urges nations to affirmatively enroll students with rural backgrounds into health professional education among the interventions for increasing HCW retention in rural settings [20]. Here, we found that pharmacists with rural childhoods were more likely to stay in their public hospital jobs than those with urban-based childhoods. Thus, the impact of social connection to rural communities appears to extend beyond retention in rural areas to retention in public hospitals.

In developing countries, remuneration for HCWs is too low to meet financial needs. The entry salary for a pharmacist in Uganda’s public hospitals is USD 345 [35]. Underpaid, HCWs cope by moonlighting and/or engagement in non-professional activities [30]. In Kenya, DR Congo, and Sierra Leone, recent studies found petty trade, farming, motorbike commuter, short-term jobs (consultancy), and part-time jobs to be common among HCWs [32, 36, 37]. We also found that majority (60%) of public hospital pharmacists are engaged in auxiliary economic activities. Besides, more pharmacists engaged in auxiliary activities were still in public service compared to those who were not. The same has been reported with medical doctors elsewhere. In Sri Lanka, doctors who have shunned emigration have cited the freedom to undertake dual practice as pull factor [34]. Unfortunately, auxiliary activities and dualism reduce the commitment of HCWs to their jobs [30]. Hence, stringent monitoring of staffs is necessary.

## Conclusions

Most pharmacists in Uganda are in dual practice. To minimize the detrimental effects of dual practice on public health, all public sector pharmacists ought to declare their side jobs to their supervisors. Meanwhile, pharmacists’ shortage in Uganda’s public hospitals is a combined result of inadequate recruitment, attraction, and retention. Thus, pharmacist shortage in Uganda’s public sector would significantly reduce if the government stepped up the hiring pace. Interventions addressing the factors strongly associated with attraction and retention should also be considered, for example, affirmative admissions of students with rural childhood and from families of low socio-economic status into pharmacy degrees and rurally oriented education. The public health sector could also benefit from raising the number of pharmacy graduates posted to countryside internships. Upgrading of public hospital pharmacists to postgraduate degrees is unfavorable to retention. Perhaps, public hospital pharmacists should be bonded during postgraduate education.

Because this study partly investigated retention in public sector, the sample was selected purposively to include as many pharmacists with a history in the public sector as possible. Therefore, the proportion of pharmacists who have ever applied for public sector jobs reported here may be higher than it actually is. Secondly, the small population of public health care pharmacists in Uganda made proportionate representation in the sample inappropriate. Thirdly, the small sample of public health care pharmacists relative to the overall sample could have affected the statistical significance of some study findings. A future study with more public health care pharmacists is recommended should their population in Uganda increase. Lastly, a study of pharmacists from low socio-economic families is required to understand its contrasting associations with interest in and rejection of public hospital jobs as found in this study.

## Additional files

Additional file 1: Desk review guide (DOCX 16 kb)
Additional file 2: Structured questionnaire (DOCX 19 kb)
Additional file 3: Hiring patterns of pharmacists into public hospitals (XLSX 12 kb)

## Abbreviations

A’Level: Advanced level certificate of education
ADEs: Adverse drug events
DR Congo: Democratic Republic of the Congo
HCW: Health care worker
HRD: Human Resource Department
HSC: Health Service Commission
IRB: Institutional Review Board
MOH: Ministry of Health
PBL: Problem-based learning
UGX: Uganda shillings
USD: United States Dollars
WHO: World Health Organization
