# Supplementary material for: Factors associated with occupancy of pharmacist positions in public sector hospitals in Uganda: a cross-sectional study
Source: Hum Resour Health. 2017 Jan 5;15:1. doi: 10.1186/s12960-016-0176-x (PMC5217537; doi:10.1186/s12960-016-0176-x)
Supplement: Additional file 1: — Desk review guide (DOCX 16 kb) [file 12960_2016_176_MOESM1_ESM.docx]

Additional file 1. Desk review guide

**Recruitment and Retention over 10 Financial Years (2004/2005-2014/2015)**

| **Financial Year** | **Type** | **Vacancies** | **Positions Filled** | **Positions Abandoned** | **Comments** |
| --- | --- | --- | --- | --- | --- |
| **2004/2005** | NRH |  |  |  |  |
|  | RRH |  |  |  |  |
|  | GH |  |  |  |  |
| **2005/2006** | NRH |  |  |  |  |
|  | RRH |  |  |  |  |
|  | GH |  |  |  |  |
| **2006/2007** | NRH |  |  |  |  |
|  | RRH |  |  |  |  |
|  | GH |  |  |  |  |
| **2007/2008** | NRH |  |  |  |  |
|  | RRH |  |  |  |  |
|  | GH |  |  |  |  |
| **2008/2009** | NRH |  |  |  |  |
|  | RRH |  |  |  |  |
|  | GH |  |  |  |  |
| **2009/2010** | NRH |  |  |  |  |
|  | RRH |  |  |  |  |
|  | GH |  |  |  |  |
| **2010/2011** | NRH |  |  |  |  |
|  | RRH |  |  |  |  |
|  | GH |  |  |  |  |
| **2011/2012** | NRH |  |  |  |  |
|  | RRH |  |  |  |  |
|  | GH |  |  |  |  |
| **2012/2013** | NRH |  |  |  |  |
|  | RRH |  |  |  |  |
|  |  |  |  |  |  |
|  | GH |  |  |  |  |
| **2013/2014** | NRH |  |  |  |  |
|  | RRH |  |  |  |  |
|  | GH |  |  |  |  |
| **2014/2015** | NRH |  |  |  |  |
|  | RRH |  |  |  |  |
|  | GH |  |  |  |  |
