# Supplementary material for: Factors associated with occupancy of pharmacist positions in public sector hospitals in Uganda: a cross-sectional study
Source: Hum Resour Health. 2017 Jan 5;15:1. doi: 10.1186/s12960-016-0176-x (PMC5217537; doi:10.1186/s12960-016-0176-x)
Supplement: Additional file 2: — Structured questionnaire (DOCX 19 kb) [file 12960_2016_176_MOESM2_ESM.docx]

Additional file 2. Study questionnaire

**Questionnaire code: ____________________________ Date: ________________________**

**Research Assistant’s Code: _______________________**

|  | **Question** | **Response(s)** | |
| --- | --- | --- | --- |
|  | 1. **Socio-demographic data** |  | |
|  | Name/Initials: |  | |
|  | Age (Please Tick Box): | ①21 – 25 years  ②26 – 30 years  ③31 – 35 years  ④36 – 40 years  ⑤ 40 years and above | |
|  | What is your gender? | ①Male  ② Female | |
|  | What best describes your marital status? | ① Single  ② Married | |
|  | What is your highest education level attained? | ① Bachelor’s Degree  ② Postgraduate Degree  ③ Master’s Degree  ④ PhD | |
|  | What mode of learning did you use on your Pharmacy degree (select only the one which best applies to you)? | ① Lecturing/teaching  ② Problem-based learning | |
|  | In which sector are you currently employed? | ①Public sector alone  ②Private sector alone  ③Non-profit alone  ④Both public and private  ⑤Both public and non-profit  ⑥Both private and non-profit  ⑦None of the above | |
|  | Geographical location of your work station | ①Kampala, Wakiso, Mukono  ②Far central Uganda  ③Eastern Uganda  ④Northern Uganda  ⑤Western Uganda | |
|  | Where did you spend most of your childhood (1-18 years) | ① Capital City  ② Other urban council (town)  ③ Rural setting | |
|  | Where are your current family located? | ① Capital City  ② Other urban council (town)  ③ Rural setting | |
|  | Have you ever applied for a public hospital pharmacist job post-internship? | ①Yes  ②No | |
|  | If you have ever applied for a public hospital pharmacist vacancy, have you ever rejected appointment as a public hospital pharmacist? | ①Yes  ②No | |
|  | If you have ever applied for a public hospital pharmacist vacancy, have you ever accepted appointment as a public hospital pharmacist? | ①Yes  ②No | |
|  | If you have ever been appointed as a public hospital pharmacist, are you still working in the public sector? | ①Yes  ②No | |
|  | 1. **Educational/Professional factors** |  | |
|  | Route through which one joined Pharmacy School | ①Direct Entry (From Secondary School)  ②Diploma/Mature Entry | |
|  | Prior to commencing your Bachelor of Pharmacy Degree had you ever worked? | ① Yes  ② No | |
|  | If Yes, in which sector had you worked? | ① Public sector  ② Private sector  ③ Nonprofit sector (NGOs, civil society) | |
|  |  |  | |
|  | In which hospital and region of the country did you do your pharmacist internship? | Hospital: ______________________________________  Region: ______________________________________ | |
|  | Where did you attain secondary education? | ① Capital City  ② Other urban council (town)  ③ Rural setting | |
|  | Where did you attain primary/elementary education? | ① Capital City  ② Other urban council (town)  ③ Rural setting | |
|  | 1. **Economic factors** |  | |
|  | How do you best describe the socio-economic status of your family at the time you were an ‘A’ Level student? | | ①Low income  ②Middle income  ③High income |
|  | How important are wages in your choice among the public sector, private sector career, and non-profit sector? | | ①Not important  ②Slightly important  ③Moderately important  ④Important  ⑤Very important |
|  | What is the minimum salary offer you accept for a fulltime pharmacist job? | | ①Any amount  ②UGX 500,000  ③UGX 500,001-750,000  ④UGX 750,001-1,000,000  ⑤UGX1,000,001-1,500,000  ⑥1,500,001-2,000,000  ⑦Above UGX 2,000,000 |
|  | If you have never applied for public hospital pharmacist job, is low pay the major deterrent? | | ① Yes  ② No |
|  | If you have ever rejected a public hospital pharmacist job offer, was low pay the major deterrent? | | ① Yes  ② No |
|  | If you have ever worked as a public hospital pharmacist but later quit, was low pay the major trigger? | | ① Yes  ② No |
|  | Besides employment, do you have alternative income sources (e.g. additional jobs, business | | ① Yes  ② No |

***Thank you so much for your co-operation***
